# Editorial: Regulation of Ubiquitination and Sumoylation Signaling in Disease

**DOI:** 10.3389/fcell.2022.970683

**Published:** 2022-07-12

**Authors:** Zhenghong Lin, Xiaohua Yan

**Affiliations:** ^1^ School of Life Sciences, Chongqing University, Chongqing, China; ^2^ Department of Biochemistry and Molecular Biology, School of Basic Medical Sciences, Nanchang University, Nanchang, China

**Keywords:** ubiquitination, sumoylation, ISGylation, neddylation, PROTAC (proteolysis-targeting chimeric molecule)

Ubiquitination/Ubiquitin-like protein modifications play an important role in the regulation of diverse physiological processes, such as DNA damage repair, cell cycle progression, cell proliferation, apoptosis and differentiation, signal transduction and gene transcriptional regulation, vesicle transport, autophagy, immunity, etc. In addition, ubiquitination and ubiquitin-like protein modifications have also been widely involved in pathological processes including metabolic disorders, inflammation, tumorigenesis, amongst others. Therefore, in recent years, it has received increasing interest to identify novel molecular targets that could lead to the development of new drugs. A deeper understanding of the ubiquitination and ubiquitin-like modifications-mediated signaling pathways and their regulatory mechanisms is urgently needed in order to identify novel molecular players and therapeutic targets for treatment of cancer and other diseases. In this Research Topic, we collected nine articles to discuss the roles of the ubiquitination/ubiquitin-like modifications in regulating diverse signal transduction pathways, thereby providing new insights to understand why and how dysregulation of them may drive pathological progression or trigger disease and aimed to provide clues for the treatment of these diseases.

The ubiquitin-proteasome system (UPS) is the main machinery contributive to the control of protein degradation in eukaryotic cells. Ubiquitination can occur under the successive actions of E1 ubiquitin-activating enzymes, E2 ubiquitin-conjugating enzymes, E3 ubiquitin-ligase enzymes, and/or E4. WW domain-containing E3 ubiquitin protein ligase 1 (WWP1) is a member of the C2-WW-HECT E3 ubiquitin ligase protein family. The Kuang et al. summarized the recent advances of WWP1 E3 ubiquitin ligase in cancer progression. They discussed the factors that cause mRNA level increase of WWP1 gene, the regulation of enzymatic activity of WWP1 protein, and its autoinhibitory mechanism in steady state.

E4B belongs to the U-box E3 ubiquitin ligase family and functions as either an E3 or an E4 enzyme in protein ubiquitination. Lu et al. studied the differential degradation of TRA2A and PYCR2 by E3 ubiquitin ligase E4B. In their study, they validated the ubiquitination of TRA2A and PYCR2 by E4B *in vitro* and in mammalian cells. They found that E4B mediated the degradation by forming K11- and K48- linked polyubiquitin chains on TRA2A and PYCR2, respectively. Intriguingly, both E4B and its substrates TRA2A and PYCR2 are overexpressed in hepatocellular carcinoma (HCC) cells, and E4B-mediated ubiquitination does not lead to protein degradation of TRA2A or PYCR2. Therefore, they concluded that other factors may exist to control the degradation of TRA2A and PYCR2 in HCC.

Primary cilia are microtubule-based, non-motile sensory organelles present in most types of growth-arrested eukaryotic cells. They are regarded as the signal transduction hubs that receive and transmit external signals to the cells, thus controlling cell growth and differentiation. Mutation of ciliary structure-related genes has been reported to cause a wide array of developmental genetic disorders. Senatore et al. summarized recent advances of primary cilia in controlling growth, differentiation and development. They discussed the interplay among UPS, autophagy and signaling pathways and concluded that they may act in synergy to control the ciliary homeostasis.

In contrast to polyubiquitination-mediated protein degradation, sumoylation, a ubiquitin-like post-translational modification, usually does not regulate protein stability but modulates signal transduction. Zhu et al. summarized the current understanding of the ubiquitination and sumoylation signaling in cellular metabolic regulation. They discussed how ubiquitination and sumoylation affect cancer metabolism by regulating the key enzymes involved in metabolic pathways to reshape metabolism and finally facilitate cancer progression.

Besides sumoylation, another ubiquitin-like post-translational modification is neddylation. It occurs *via* the activation of the neural precursor cell expressed, developmentally downregulated protein 8 (NEDD8) by three enzymes: an activating enzyme, a conjugating enzyme, and finally a ligase. Zhu et al. summarized the recent advances of the association between neddylation and immune response. They discussed the importance of NEDD8 in innate and adaptive immune cells and its regulatory role in the anti-viral pathways. Finally, they reminded us that the neddylation inhibitor MLN4924, as an anti-tumor medicine, has negative effects in immunity and need to take careful consideration when it is clinically used.

Apart from sumoylation and neddylation, ISGylation represents another ubiquitin-like post-translational modification. Zhang et al. summarized how ISGylation, especially ISG15, functions in innate antiviral immunity and pathogen defense responses. Through covalent binding with the host and viral target proteins, ISG15 inhibits the release of viral particles, hinder viral replication, and regulates the incubation period of viruses, thereby exerting strong antiviral effects.

Protein post-translational modification by ubiquitin is a reversible biochemical process. Deubiquitinating enzymes (DUBs) are responsible for removing ubiquitin or ubiquitin-like modifications from substrate proteins, thereby gaining increasing attention. Li et al. summarized the multiple functions of the deubiquitinase USP13 and its target inhibition. They discussed the structure and function of USP13 and its actions in various human diseases, in addition to the development of inhibitors. They hoped to provide some enlightenment for drug development and therapy of USP13-caused malignant diseases. Of relevance, Rossi and Rossi summarized the roles of USP19 in oncogenesis and cancer progression. They reviewed the current knowledge of USP19 as to the control of several cellular processes in different neoplasms, which highlights a complexity of USP19 function which possesses both positive and negative regulation activities in tumorigenesis and cancer progression. They suggested that USP19 might represent a novel putative pharmacologic target in oncology, underscoring the potential of identifying specific modulators to test in clinical settings.

Ubiquitination, as one of the most important post-translational events, is a dynamic process primarily responsible for protein degradation *via* proteasomes. Importantly, ubiquitination can be targeted for the treatment of human disease. Proteolysis-targeting chimera (PROTAC) is a recently emerged technique that has great potential to be clinically used in the treatment of cancer. Lospinoso Severini et al. summarized the recent advances of the PROTAC strategy as therapeutic option in glioblastoma. They discussed the advantages and limitations of PROTAC development and safety considerations for their application in clinical usage.

Together, understanding the biochemical nature and biological functions of protein post-translational modifications, especially ubiquitination and ubiquitination-like modifications, is of great significance in unravelling the molecular mechanisms underlying the development of human diseases, such as cancer. In addition, this is also a prerequisite for discovering new molecular targets and developing novel anti-cancer drugs.

